# Microwave-Assisted Chitosan-Functionalized Graphene Oxide as Controlled Intracellular Drug Delivery Nanosystem for Synergistic Antitumour Activity

**DOI:** 10.1186/s11671-021-03525-y

**Published:** 2021-04-30

**Authors:** Mengjun Shu, Feng Gao, Min Zeng, Chulang Yu, Xue Wang, Renhua Huang, Jianhua Yang, Yanjie Su, Nantao Hu, Zhihua Zhou, Ke Liu, Zhi Yang, Hongtao Tan, Lin Xu

**Affiliations:** 1 Key Laboratory of Thin Film and Microfabrication (Ministry of Education), Department of Micro/Nano Electronics, School of Electronic Information and Electrical Engineering, Shanghai Jiao Tong University, Shanghai, 200240 People’s Republic of China; 2 Key Laboratory of Hepatosplenic Surgery (Ministry of Education), Department of General Surgery, The First Affiliated Hospital of Harbin Medical University, Harbin, 150001 People’s Republic of China; 3 Department of Ophthalmogy, Shanghai General Hospital (Shanghai First People’s Hospital), School of Global Health, Chinese Center for Tropical Diseases Research, Shanghai Jiao Tong University School of Medicine, Shanghai Eye Disease Prevention and Treatment Center/Shanghai Eye Hospital, National Clinical Research Center for Eye Diseases, Shanghai Key Laboratory of Ocular Fundus Diseases, Shanghai Engineering Center for Visual Science and Photomedicine, Shanghai, 200080 People’s Republic of China; 4 Department of Dermatology, Shanghai Ninth People’s Hospital, Affiliated To Shanghai Jiao Tong University School of Medicine, Center for Specialty Strategy Research of Shanghai Jiao Tong University China Hospital Development Institute, Shanghai, 200011 People’s Republic of China; 5 State Key Laboratory for Managing Biotic and Chemical Threats To the Quality and Safety of Agro-Products, Key Laboratory of Biotechnology in Plant Protection of MOA and Zhejiang Province, Institute of Plant Virology, Ningbo University, Ningbo, 315211 People’s Republic of China; 6 Department of Radiation, Renji Hospital, School of Medicine, Shanghai Jiao Tong University, Shanghai, 200240 People’s Republic of China

**Keywords:** Graphene oxide, Drug delivery, Adriamycin, Microwave-assisted reduction, Breast cancer, HER2, Trastuzumab

## Abstract

To achieve better antitumour efficacy, it is urgent to improve anticancer drug delivery efficiency in targeting cancer cells. In this work, chitosan-functionalized graphene oxide (ChrGO) nanosheets were fabricated via microwave-assisted reduction, which were employed to the intracellular delivery nanosystem for anticancer drug agent in breast cancer cells. Drug loading and release research indicated that adriamycin can be efficiently loaded on and released from the ChrGO nanosheets. Less drug release during delivery and better biocompatibility of ChrGO/adriamycin significantly improve its safety and therapeutic efficacy in HER2-overexpressing BT-474 cells. Furthermore, ChrGO/adriamycin in combination with trastuzumab exhibited synergistic antitumour activity in BT-474 cells, which demonstrated superior therapeutic efficacy compared with each drug alone. Cells treated with trastuzumab (5 μg/mL) or equivalent ChrGO/adriamycin (5 μg/mL) each elicited 54.5% and 59.5% cell death, respectively, while the combination treatment with trastuzumab and ChrGO/adriamycin resulted in a dramatic 88.5% cell death. The dual-targeted therapy displayed higher apoptosis, indicating superior therapeutic efficacy due to the presence of different mechanisms of action. The combined treatment of ChrGO/adriamycin and trastuzumab in BT-474 cells induced cell cycle arrest and apoptosis, which ultimately led to the death of augmented cancer cells. This work has provided a facile microwave-assisted fabrication of ChrGO as a controlled and targeted intracellular drug delivery nanosystem, which is expected to be a novel promising therapy for treating HER2-overexpressing breast cancer cells.

**Supplementary Information:**

The online version contains supplementary material available at 10.1186/s11671-021-03525-y.

## Introduction

The HER2 receptor is a member of the EGFR receptor family that mediates cancer cell growth and differentiation and is highly overexpressed in 20–30% of human breast cancers, leading to a metastatic tumour phenotype and poor prognosis [1]. HER2 is also overexpressed in approximately 20% of human gastric cancers [2]. Trastuzumab, a humanized monoclonal therapeutic antibody, demonstrates promising therapeutic advantages as the first-line therapy in HER2-overexpressing breast cancer patients [3]. However, the overall response rate to trastuzumab remains modest: 15–30% when treated as a single therapy and 50–75% when used in combined treatment with chemotherapy drugs [4]. Among those patients who do respond to trastuzumab, a majority of them eventually progress following initial response and acquire resistance overtime after continuous treatment [5]. Thus, it is essential and necessary to develop additional novel therapies for HER2-overexpressing patients to improve overall survival rates.

Anthracyclines still serve as the backbone of cancer treatment. For the anthracycline agents, the topoisomerase II inhibitor adriamycin has been widely used to treat many cancers, such as breast, lung and lymphoid cancers [6]. Adriamycin has effective efficacy in the treatment of HER2-overexpressing breast cancer patients, due to the proximity between the HER2 gene and the topoisomerase II gene [7]. Despite the clinical benefit observed with anthracyclines-based therapies in breast cancer, cardiac dysfunction has restricted more expansive therapeutic applications. In the clinical trial, trastuzumab in combination with adriamycin demonstrated significant efficacy, while highly dose-dependent cardiotoxicity was a problem that has to be solved [8]. Sustained release of chemotherapy agents at the tissue of cancer target is recognized as a good solution to lower doses required for therapeutic efficacy and improve the safety profiles [9]. To achieve better antitumour efficacy but fewer side effects, there is an urgent need to improve anticancer drug delivery efficiency in targeting cancer cells.

**Scheme 1:**
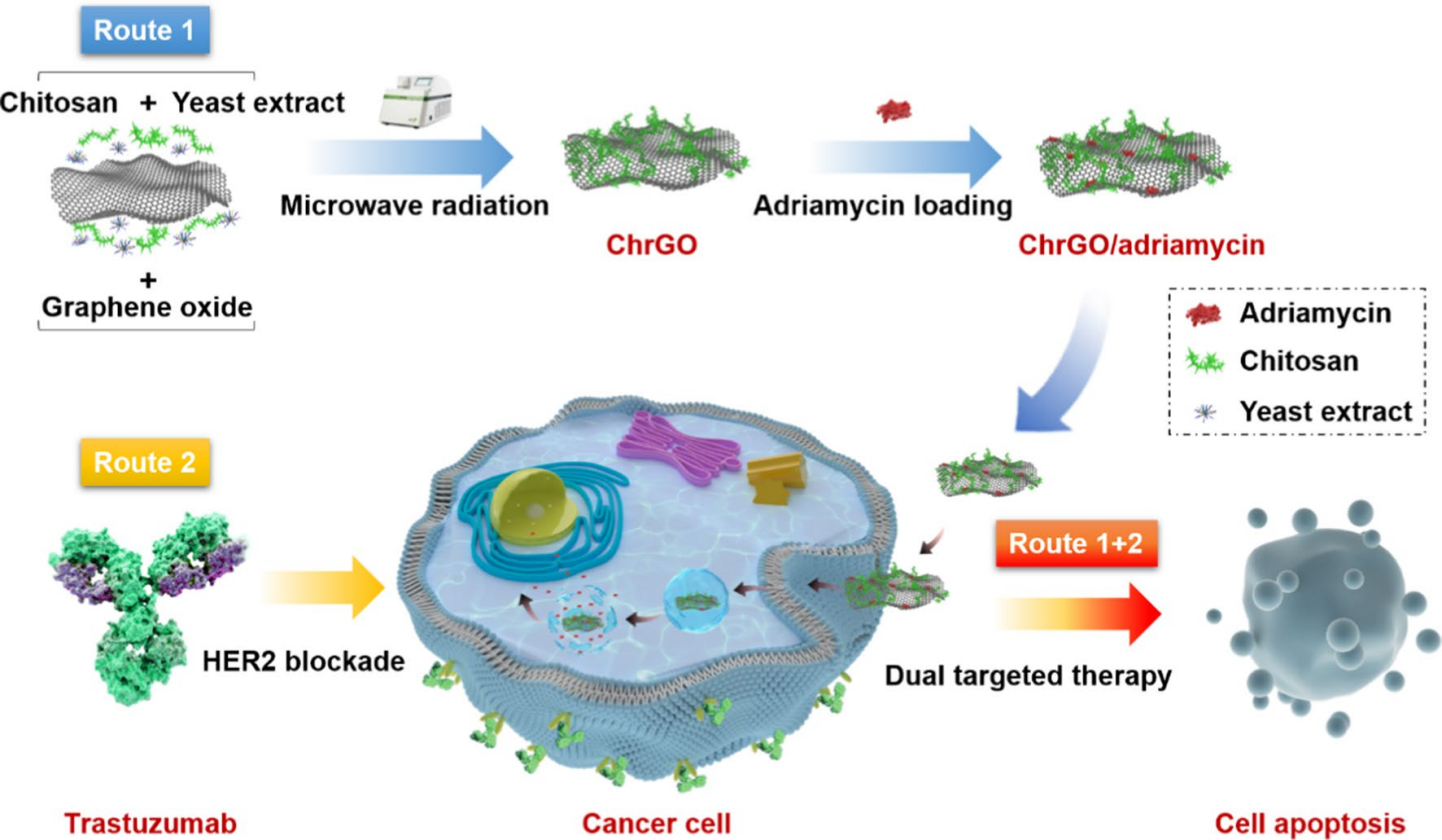
Microwave-assisted biofunctionalization and reduction of graphite oxide as controlled drug delivery nanosystem for dual-targeted therapy in HER2-overexpressing BT-474 cells

Graphene oxide (GO)-based nanomaterials have displayed great promise in the controlled delivery of chemotherapy agents [10]. Numerous studies have been demonstrated that the toxicity of GO is associated with its surface functionalization [11]. Recently, environmentally friendly agents, such as chitosan and PEG, have been reported to functionalize of GO with less poisonous surface functional group [12, 13]. Nanoparticle colloidal stability in biological media is crucial for the development of effective and safe drug delivery system for clinical use [14]. Functionalized GO nanosheets have gained great attention in biomedical applications owing to their properties such as biocompatibility and stability. As an excellent candidate for graphene-based biomaterial, colloidal stability of GO or rGO is important for controlling the performance of drug carriers. The poly (ethylene glycol)-functionalized GO exhibited an improved colloidal property in cell culture media [15]. P. Khanra reported a novel method for the simultaneous reduction and bio-functionalization of GO by using yeast cells as reductive biocatalyst [16, 17]. Avinav G presented a one-pot biosynthesis of GO by utilizing yeast extract during an autoclave process [18]. Sonication treatment of micrometer-sized GO sheets for hours resulted in GO nanosheets, which showed higher colloidal stability compared to regular micrometer-sized GO [19]. Although the previous studies demonstrated the high potential of GO for biomaterials, the bio-functionalized GO nanosheets with chitosan by microwave-assisted reduction using cell-free yeast extract have not been studied until now. For this purpose, a novel chitosan-functionalized graphene oxide (ChrGO) structure was constructed by a facile microwave synthesis system that utilizes yeast extract as the reductant. Moreover, the effective functionalization of GO with biocompatible chitosan provides a potential platform for efficient drug loading and delivery. Drug loading and release studies demonstrated that adriamycin was efficiently loaded on and released from the ChrGO nanosheets. The ChrGO/adriamycin composites showed significant antitumour activity in a concentration-dependent manner. Particularly, the combinational treatment with ChrGO/adriamycin and trastuzumab resulted in an enhanced growth inhibition effect in BT-474 cells compared to monotherapy. The synergetic antitumour efficacy of these agents was revealed to be mediated through cell cycle arrest and apoptosis, which ultimately led to cancer cell death. This work reported a promising route for the rapid and cost-effective production of ChrGO composites, which delivered chemotherapy agents in a spatiotemporally controlled manner for efficient cancer treatment (Scheme 1).

## Materials and Methods

### Materials

Graphite powder was obtained from Qingdao Huatai Tech (Qingdao, China). Chitosan and adriamycin were purchased from Aladdin Co., Ltd. (Shanghai, China). Trastuzumab was purchased from Hoffmann-La Roche Ltd (Basel, Switzerland). Roswell Park Memorial Institute-1640 (RPMI-1640), Dulbecco’s modified Eagle medium (DMEM) and fetal bovine serum (FBS) were purchased from Invitrogen Corporation (Camarillo, USA). Amicon^®^ Ultra centrifugal filters were purchased from Merck Millipore. CellTiter 96^®^ aqueous one solution cell proliferation assay kit and Caspase-Glo^®^ 3/7 assay system kit were obtained from Promega Corporation (Madison, USA). Dry baker’s yeast was obtained from AB/Mauri Co., Ltd. BT-474 and Cos-7 cell lines were obtained from the Cell Bank of Chinese Academy of Sciences (Shanghai, China). Luria–Bertani medium was purchased from Sangon Biotech Co., Ltd. All chemicals were analytical grade and commercially available without further purification.

### Microwave-Assisted Reduction of Chitosan-Functionalized GO

Graphite oxide (GO) was prepared from native graphite flakes using a modified Hummer’s method [14]. To obtain a single layer of nano-sized GO, the GO was exfoliated with an ultrasonic probe (Scientz, China) operating at 800 W for 8 h. Finally, the exfoliated GO was dispersed in deionized water for further use. The partially reduced chitosan–GO (ChrGO) nanosheets were synthesized through microwave-assisted reduction of GO with chitosan aqueous solution using a microwave synthesis system. The cell-free yeast extract was used for the biosynthesis of ChrGO via microwave-assisted reduction. First, the stocked yeast cells were activated by inoculation into Luria–Bertani medium and shaking at 135 rpm for 18 h at 25 °C. The activated yeast cells were transferred into 2% sucrose solution shaking at 135 rpm for another 6 h at 25 °C. Next, 6 mL of cell-free yeast extract was obtained by centrifugal separation at 2000 rpm for 5 min. Then, 50 mg of chitosan was dissolved in 25 mL of 2% (v/v) acetic acid solution and mixed with yeast extract [20]. A 5 mg GO solution was added under vigorous magnetic stirring. Finally, the as-prepared solution was transferred into NOVA-2S microwave synthesis equipment (PreeKem Scientific Instruments, China) for the microwave reaction. The heating scheme for the microwave system involved heating to 80 °C for 5 min and holding the temperature for another 5 min. The obtained ChrGO was purified with a 100-kDa MWCO filter (Millipore, USA) and freeze-dried for further use.

### Characterizations

Transmission electron microscopy (TEM) images of nano-sized GO were obtained on a JEM-2100 transmission electron microscope with an accelerating voltage of 200 kV (JEOL, Japan). Ultraviolet–visible (UV–Vis) spectra were obtained using a UV/Vis/NIR spectrophotometer Lambda 950 (Perkin-Elmer, USA). Fourier transform infrared (FTIR) spectra were collected by using a Vertex 70 FTIR spectrometer scanning from 4000 to 400 cm^−1^ with samples prepared as KBr pellets (Bruker, Germany). Raman spectra were recorded using a Senterra R200-L Raman microscope with an excitation wavelength of 532 nm (Bruker Optics, Germany). X-ray diffraction (XRD) patterns were examined by a Bruker D8 Advance diffractometer (Bruker, Germany). The surface elements were recorded using X-ray photoelectron spectroscopy (XPS) Kratos AXIS Ultra DLD with monochromatic Al Ka radiation (1486.6 eV) (Shimadzu, Japan). Thermogravimetric analysis (TGA) was conducted on a PerkinElmer Pyris 1 TGA at a heating rate of 5 °C/min from 30 to 800 °C in a nitrogen atmosphere (PerkinElmer, USA). The surface charge of the composites was measured by a Malvern Zeta Nano ZS-90 instrument (Malvern, UK).

### Drug Loading and Releasing

Four milligrams of ChrGO nanoparticles was suspended in 20 mL of adriamycin aqueous solution (0.4 mg/mL). After sonication for 0.5 h, stirring was performed in the dark for 24 h, avoiding light. The unloaded adriamycin was removed by centrifugation filtration through 50-kDa MWCO Amicon filters (Millipore, USA) and washed away with phosphate-buffered saline (PBS, pH 8.0) until the supernatant turned colourless. The concentration of adriamycin was determined using a standard adriamycin concentration curve by UV–Vis spectrometry at 490 nm with a SpectraMax^®^ M5 microplate reader (Molecular Devices, USA). The amount of adriamycin loading onto ChrGO was determined with a UV–Vis absorbance by measuring the concentration of the loss of adriamycin in the supernatant of Amicon^®^ Ultra-15 mL centrifugal filter.

The adriamycin release characteristics of ChrGO were studied. The adriamycin-loaded ChrGO was submerged in 10 mL of PBS buffer solution. At specified intervals, 2 mL of released adriamycin solution detached from ChrGO was collected by centrifugation filtration through 50-kDa MWCO Amicon filters (Millipore, USA). The volume of ChrGO/adriamycin solution was kept constant by adding 2 mL of fresh PBS buffer solution after each sampling. The amount of adriamycin released from ChrGO was measured by a UV–Vis absorbance at 490 nm by a SpectraMax^®^ M5 microplate reader (Molecular Devices, USA). The release studies were investigated in different pH solutions (pH values 5 and 7.4).

### Biocompatibility Analysis and Therapeutic Efficacy Assay

BT-474 and Cos-7 cells were maintained in RPMI-1640 supplemented with 10% FBS or DMEM supplemented with 10% FBS, respectively, and cultured in a humidified atmosphere of 5% CO_2_ at 37 °C. The biocompatibility assay of GO and ChrGO was performed on BT-474 or Cos-7 cells via cell cytotoxicity assay. Cells were plated into 96-well flat plates at a density of 3 × 10^4^ cells per well and preincubated for 18 h before treatment with GO and ChrGO. Then, the dilutions of the tested agents were added to the cells and incubated for another 24 h. The viability of the cells was determined by CellTiter 96^®^ aqueous one solution. The absorbance was measured at 490 nm by a SpectraMax^®^ M5 microplate reader (Molecular Devices, USA).

The efficacy of ChrGO/adriamycin complexes alone or in combination treatment with trastuzumab on proliferation in BT-474 cells was investigated by proliferation inhibition assay [16]. BT-474 cells were plated into 96-well flat plates at a density of 1 × 10^4^ cells per well and incubated for 24 h. Then, ChrGO/adriamycin complexes alone or in combined treatment with trastuzumab were introduced to the BT-474 cells in the culture medium. After 96-h incubation, the viable cells were determined by CellTiter 96^®^ aqueous one solution. The absorbance was measured with a SpectraMax^®^ M5 microplate reader at 490 nm (Molecular Devices, USA). The data were analysed with one-way ANOVA. *p* < 0.05 was considered statistically significant.

### Cell Cycle Analysis and Apoptosis Assay

For cell cycle analysis, BT-474 cells were plated into six-well plates at a density of 5 × 10^5^ cells per well and allowed to adhere for 16 h. The cells were treated with ChrGO/adriamycin complexes alone or in combination with trastuzumab for 24 h. BT-474 cells were harvested and fixed with 70% (v/v) ethanol at 4 °C for 24 h. The fixed BT-474 cells were stained with propidium iodide solution (15 μg/mL) containing ribonuclease A (10 μg/mL) at 25 °C for one hour. Then, the cells were analysed with a flow cytometer (BD Biosciences, USA). For the apoptosis assay, BT-474 cells were seeded into white-walled 96-well plates at a density of 2 × 10^4^ cells per well and allowed to adhere for 18 h. The cells were treated with ChrGO/adriamycin complexes, trastuzumab alone or in combination treatment for 24 h. Then, the culture medium was discarded, and 100 µL of Caspase-Glo^®^ 3/7 reagent was added to each well and incubated at RT for 2 h. Luminescence was measured by a SpectraMax^®^ M5 microplate reader (Molecular Devices, USA). The results were analysed by one-way ANOVA. *p* < 0.05 was considered statistically significant. All studies were performed in accordance with relevant guidelines and regulations. 

## Results and Discussion

### Synthesis and Characterization

**Fig. 1:**
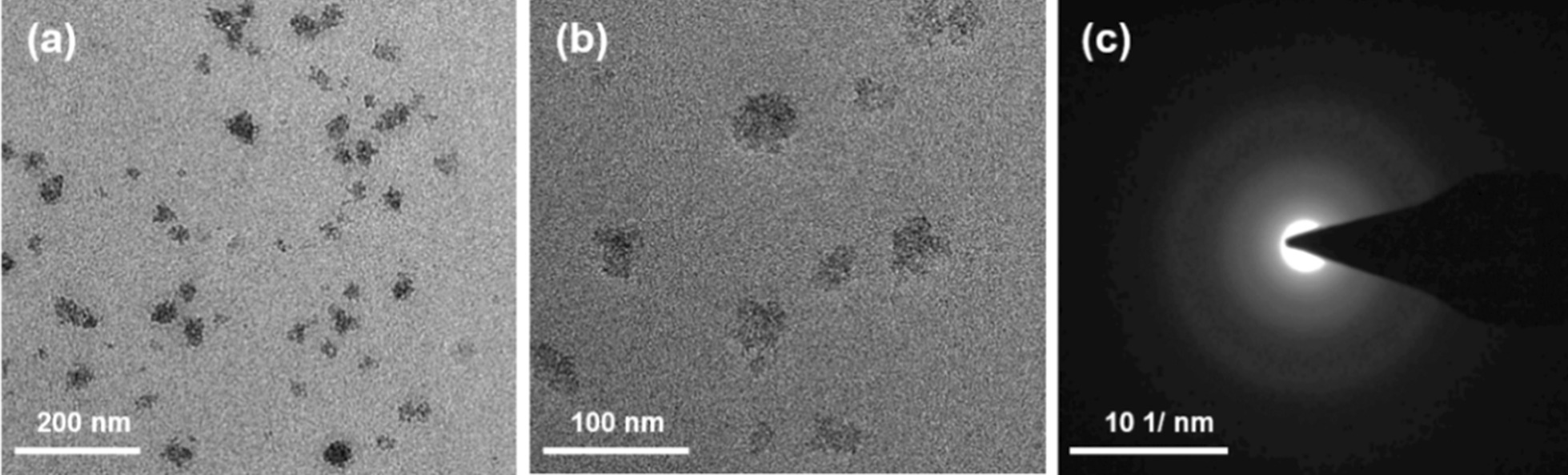
**a** Low-magnification TEM image of GO nanosheets, **b** high-resolution TEM image of nanosheets and **c** a typical SAED pattern of GO nanosheets

The morphology of the as-synthesized GO sheets was characterized by transmission electron microscopy (TEM). Figure 1a reveals the uniform size distribution of GO nanosheets below 100 nm, with an average size of approximately 45 nm. The high electron density of GO exhibited better contrast compared to chitosan, which is barely visible due to its low electron density and hydrated nature [21]. Due to the higher surface area, nanoscale GO or reduced GO sheets have been widely used as drug carriers [22]. The selected-area electron diffraction (SAED) pattern in Fig. 1c displays the concentric rings, which demonstrates the presence of a polycrystalline nature corresponding to graphene oxide [23].

**Fig. 2:**
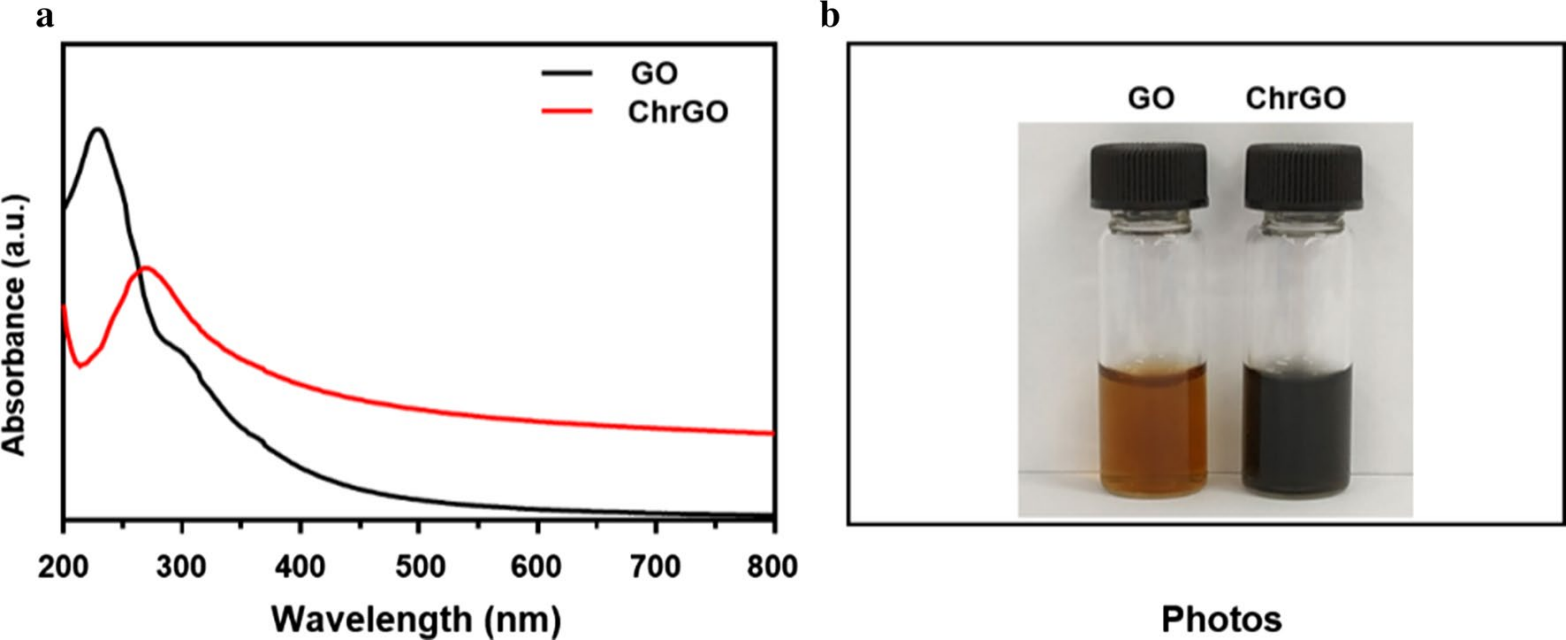
**a** UV–Vis spectrum of the GO nanosheets and ChrGO in aqueous solution, **b** photographs of GO nanosheets and synthesized ChrGO

To stabilize GO nanosheets in physiological solution, chitosan-functionalized GO nanosheets were prepared by microwave-assisted reduction using a microwave system [24]. The resulting reduced GO-chitosan (ChrGO) was analysed by UV–Vis spectrometry. Figure 2a shows the UV–Vis spectra of GO and ChrGO. GO exhibited a sharp absorption peak at 230 nm and a shoulder at 300 nm, which is ascribed to the *π*–π* transition of aromatic C=C bonds and *n*–π* transition of C=O bonds, respectively [25, 26]. After chitosan was grafted to GO, the peak at 230 nm redshifted to 270 nm for ChrGO, and the shoulder at 300 nm obviously disappeared, which was ascribed to the partial restoration of electronic conjugation among the aromatic carbon atoms [27]. A black solution of synthesized ChrGO is shown in Fig. 2b, which indicates the formation of partially reduced graphene oxide (p-rGO). Both the GO nanosheets and ChrGO were well dispersed in deionized H_2_O. The colloidal stability of GO or GO derivatives in aqueous solution is very important for their biomedical application. The results suggested that the chitosan was conjugated with GO after the reduction.

**Fig. 3:**
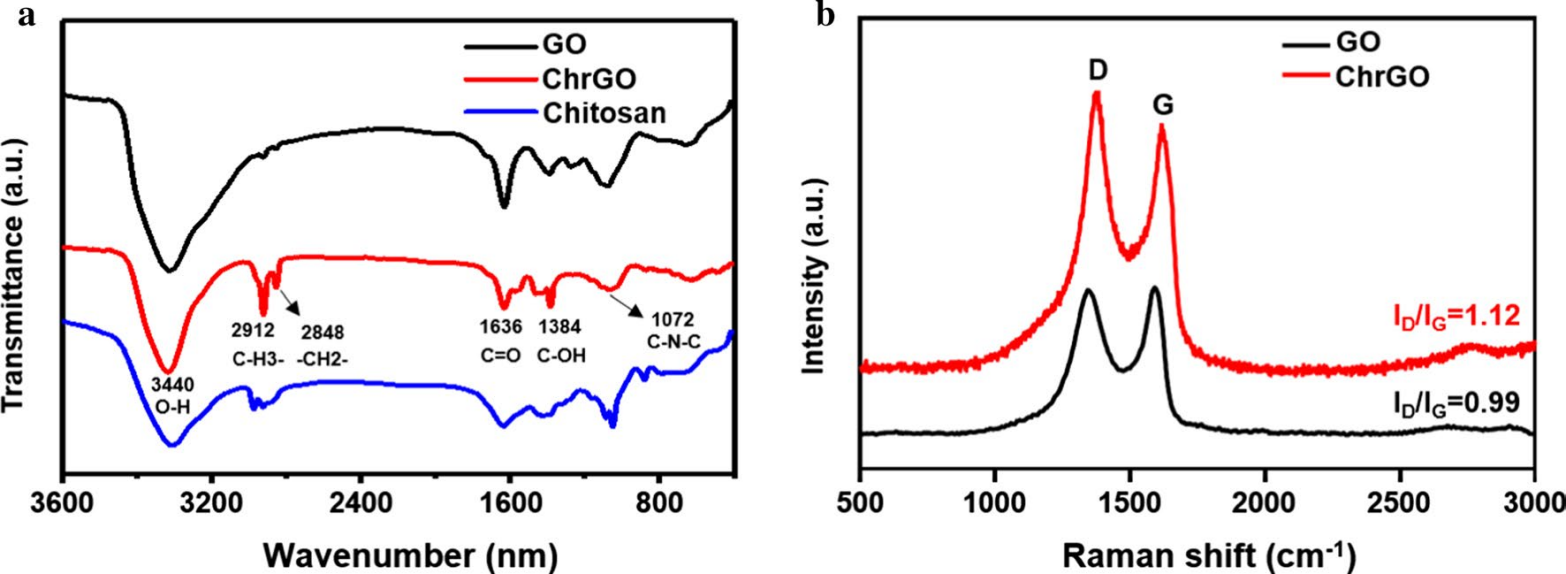
**a** FTIR spectra of GO, ChrGO and chitosan, **b** Raman spectra of GO and ChrGO

FTIR spectroscopy was performed to characterize the structure of GO, chitosan and ChrGO (Fig. 3a). The characteristic peaks of GO were situated at 3440 cm^−1^ and 1376 cm^−1^, corresponding to O–H and C–OH bonds, respectively. The peak at 1072 cm^−1^ was assigned to the stretching vibration of C–N–C. Noticeably, the peaks of chitosan molecules at 2912 cm^−1^ and 2848 cm^−1^ were attributed to the stretching vibrations of the CH_3_– and –CH_2_– [28, 29]. Functionalized p-rGO with chitosan resulted in new bonds, which led to new peaks in the ChrGO spectra. There is a significant decrease in the intensities of the C=O band at 1636 cm^−1^ in ChrGO compared to that in GO, which suggests that the reduction process removed the oxygen-containing groups of GO [30]. The FTIR spectrum of ChrGO confirmed the successful conjugation of chitosan on GO. In addition, Raman spectroscopy was used to characterize the electronic properties and structure of graphene. The G band is ascribed to the *E*_2g_ phonons of *sp*^2^ carbon domains, whereas the D band is assigned to the vibration of *sp*^3^ carbon atom domains and disordered carbon atoms. The intensity of the D band indicates the characteristics of disorders and defects in carbon sheets [31]. The reduction of GO nanosheets was also analysed in the signal ratio of the D versus G band [32]. The representative Raman spectrum of GO shows two characteristic peaks of the D band (1341 cm^−1^) and the G band (1591 cm^−1^) in Fig. 3b. Change in the relative intensity of I_D_/I_G_ value illustrates the change in the electronic conjugation state of the GO in the reduction process [33]. The shifting of the D band displays successful functionalization of reduced GO. The value of *I*_D_/*I*_G_ increases from 0.99 (GO) to 1.12 (ChrGO), which is ascribed to the introduction of *sp3* defects after functionalization and incomplete recovery of the graphene characteristic structure [34]. This observation is in good agreement with the previous report and suggests the formation of chitosan-functionalized graphene [16].

**Fig. 4:**
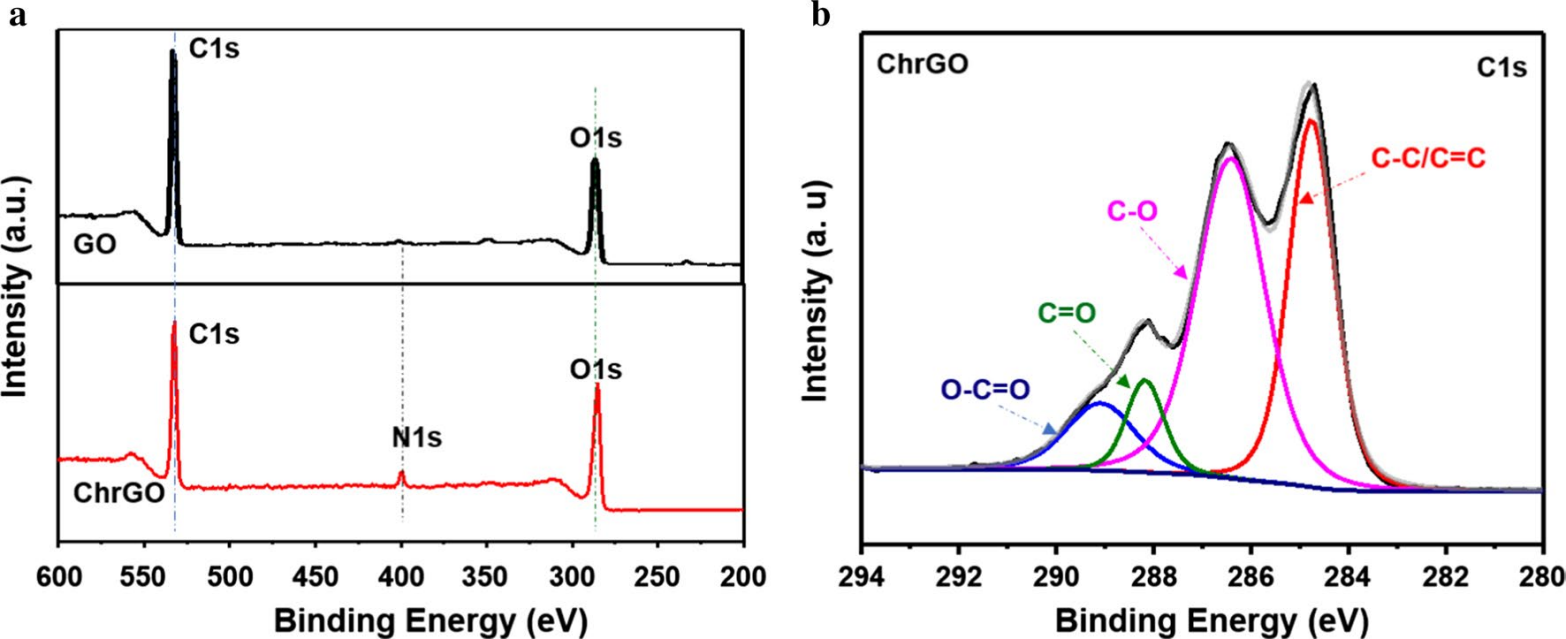
**a** Survey XPS spectra of GO and ChrGO, **b** high-resolution spectra of C1s

The surface composition of ChrGO was confirmed by XPS. The full scan of the XPS spectrum of ChrGO shows three peaks at 284, 399 and 533 eV, which are assigned to C1s, N1s and O1s (Fig. 4a), respectively. The relative elemental analysis displayed an increase in the ChrGO oxygen and nitrogen levels, with an associated decrease in the carbon content compared to GO. The high-resolution C1s spectra of ChrGO shown in Fig. 4b exhibit four separated peaks, corresponding to C–C or C=C (*sp*^2^, 284.7 eV), C-O (epoxy/hydroxyls, 286.4 eV), C=O (*sp*^3^, 288.2 eV) and O=C–O (carboxylates, 289.1 eV) bonds. The appearance of an amide peak in ChrGO provides evidence for the functionalization of chitosan on GO [35]. The abundant hydrophilic functional groups on the surface made ChrGO highly soluble in an aqueous solution, which is consistent with the FTIR results. The biomolecules such as reductive amino acids and alpha-linolenic acid in the yeast extract may have a significant role in the microwave-assisted fabrication of ChrGO [20].

**Fig. 5:**
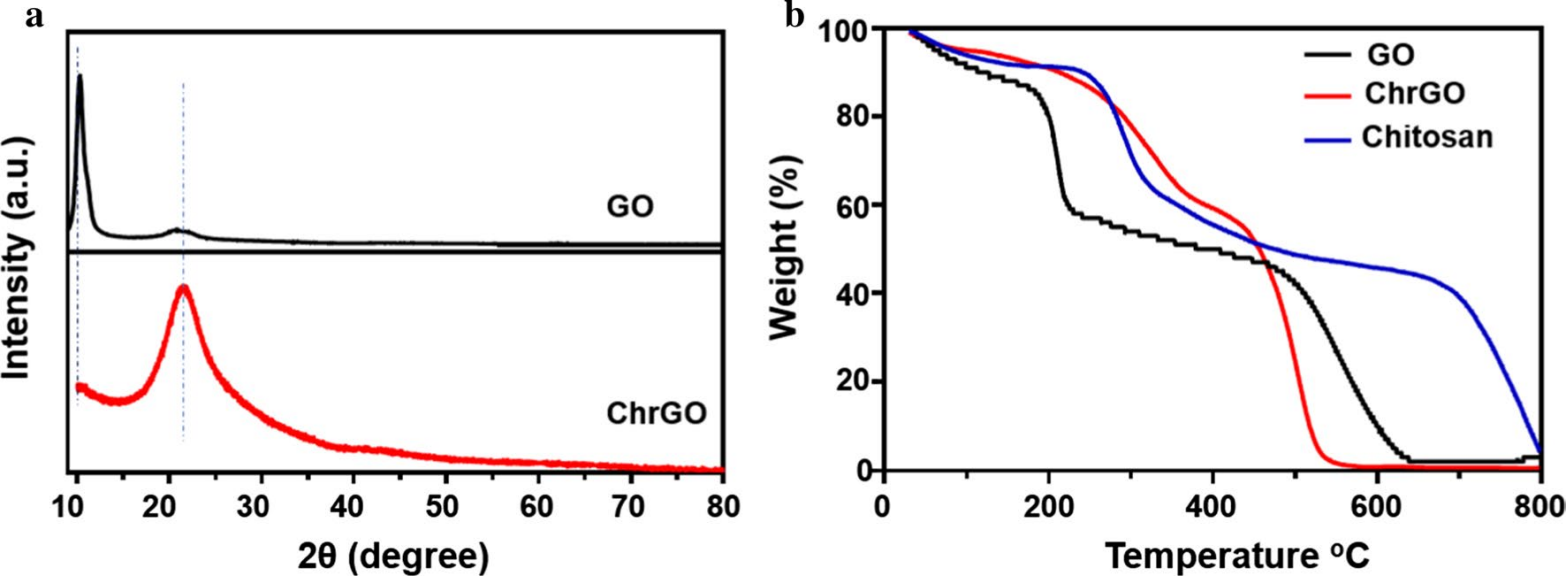
**a** XRD patterns of GO and ChrGO, **b** TGA curves of GO, chitosan and ChrGO

The X-ray diffraction (XRD) pattern of GO and ChrGO is presented in Fig. 5a. In GO XRD spectra, the major peak at 11.0° and weak peak at 20.7° clearly present the graphite structure. The feature diffraction peak at 11.0° corresponding to the (001) plane of GO, which shows the successful synthesis of GO [36]. The small bump between 20° and 24° shows the graphitic moieties, which is ascribed to the unoxidized pristine graphite [37]. With the functionalization of chitosan, the (001) peak of GO disappears, whereas the broad diffraction peak at 21.4° becomes prominent. This shift can be attributed to the reduction of the GO, where the reduction makes the rGO pack tighter than the GO. The (002) reflection of ChrGO sample is very broad, suggesting that the sample is very poorly ordered along the stacking direction, which may be ascribed to the incomplete reduction of GO [33]. The decomposition behaviours of GO, chitosan and ChrGO have been studied by thermal gravity analysis (TGA) [38]. The TGA curves of ChrGO, GO and chitosan are shown in Fig. 5b, which were measured in a nitrogen atmosphere. GO started to lose weight below 100 °C, which was attributed to the elimination of adsorbed free water in the stacked structure [39]. The GO lost 42% of its weight in the range of 191–231 °C, which was related to the decomposition of labile oxygen-containing groups. The rate of weight loss of ChrGO at 100–250 °C is significantly lower than GO, and it is obvious that ChrGO showed a different decomposition pattern as compared to GO. The thermal elimination of ChrGO and pure chitosan took place from 250 to 440 °C, which is related to the depolymerization and pyrolysis of more stable functional groups, such as glycosidic units of chitosan and carboxyl groups [40]. Compared with GO, ChrGO was thermally stable and gave a major weight loss of 36% in the first decomposition stage at 250–440 °C, whereas little weight loss is displayed for the GO. The significant weight loss in the high-temperature region of 450–800 °C was due to the thermal degradation of the carbon skeleton, which could be ascribed to the chitosan residues and biomolecules from yeast extract such as amino acids [41].

The surface charge of GO and ChrGO was measured by a Malvern Zeta Nano ZS-90 instrument, which is an important parameter of colloidal stability. The higher surface charge density on GO nanosheets creates a more stable colloidal dispersion [42]. As shown in Additional file 1: Fig. S1, the zeta potential of GO decreased monotonically from − 10.7 mV at pH 3 to − 35.5 mV at pH 11, which confirmed the negative charge on the surface of the GO nanosheets. The zeta potential value for ChrGO was comparatively less than the potential of GO at any pH ranging from 3 to 11. ChrGO nanosheets displayed stability over the entire range of pH values, while reduced GO colloids were reported to be less stable in deionized water due to increased *π*–*π* stacking in the deoxygenated surfaces [43]. Although some free amine groups were present on the surface of chitosan [44], a higher zeta potential of ChrGO was not achieved. The lower zeta potential value of ChrGO could be ascribed to the abundant negative amino acid molecules from the yeast extract, which enhanced the colloidal stability in physiological solution [20]. The negatively charged surface of ChrGO makes them potentially applicable for the loading and delivery of the drug.

### Biocompatibility of As-Synthesized ChrGO

**Fig. 6:**
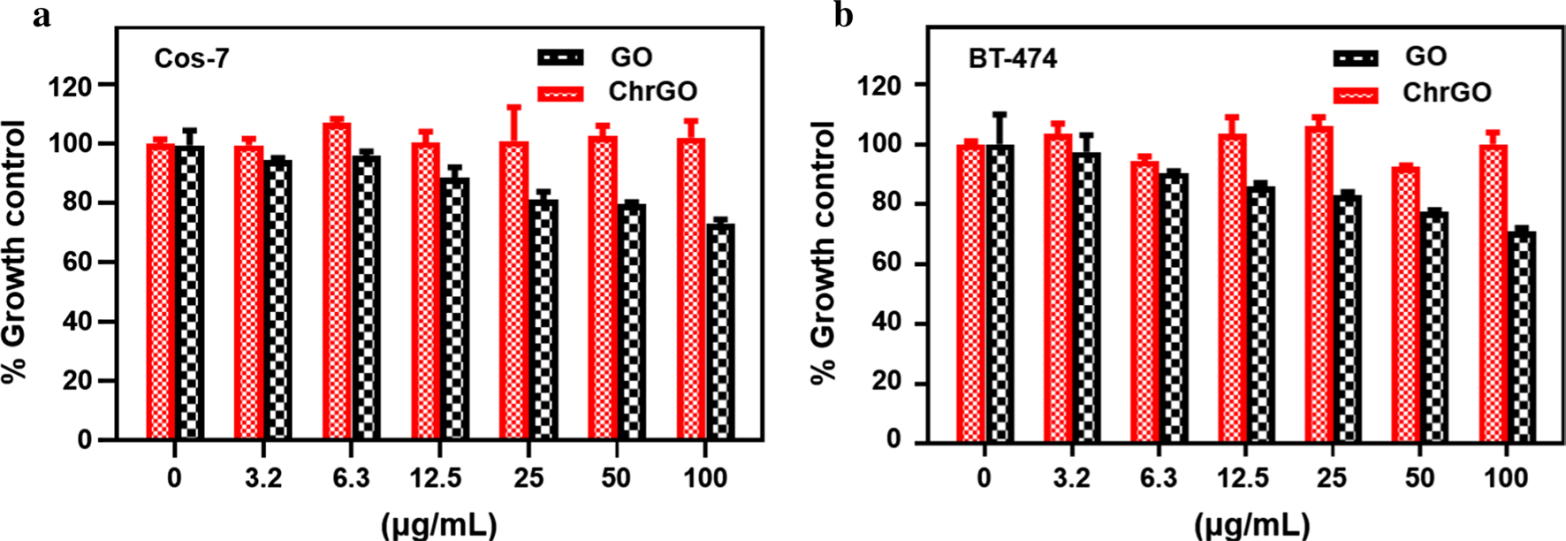
Biocompatibility of GO and ChrGO. Varying concentrations of nanoparticles were cultured with **a** Cos-7 cells and **b** BT-474 cells, and their effect on cell viability was determined

The biocompatibility of as-synthesized ChrGO is important for its applications in drug delivery. The cell cytotoxicity of ChrGO and GO was investigated by cytotoxicity assay. Cos-7 cells and BT-474 cells were incubated in the presence of either ChrGO or GO for 24 h. The results of cell cytotoxicity are shown in Fig. 6. It could be concluded that ChrGO showed no obvious cell cytotoxicity to Cos-7 and BT-474 cells. Even at a concentration of 100 μg/mL, the cell viability was above 90%. However, GO exhibited significant cell cytotoxicity in a dose-dependent manner, with only 73.0 ± 0.5% and 71.0 ± 0.5% cell survival for Cos-7 and BT-474 cells at a concentration of 100 μg/mL, respectively. The results demonstrated that chitosan functionalized on the surface of GO showed low cytotoxicity greatly and improved biocompatibility. Surface functionalization of GO with macromolecules has been reported to remarkably attenuate its cytotoxic effects [45]. Hu et al. reported that fetal bovine serum in cell medium markedly decreased the cell cytotoxicity of GO in non-small cell lung cancer A549 cells [46].

### Loading and Release of drug

The potential biomedical application of ChrGO as a drug carrier is measured by the drug loading and release behaviour. The structural characteristics of graphene derivatives are highly effective for aromatic drug delivery due to their very large specific surface area. Adriamycin is one of the primary tumour chemotherapeutics against a variety of haematologic malignancies and solid tumours and is frequently used as a model drug to evaluate the graphene derivative drug delivery systems [47]. The loading capacity of adriamycin onto ChrGO nanosheets was verified by the characteristic UV–Vis absorbance peak of adriamycin at 490 nm. As expected, the maximum loading efficiency of adriamycin bound onto ChrGO nanosheets was as high as 169.8%, which was partially attributed to the large surface area of the ChrGO nanosheets. The adriamycin-loaded ChrGO/adriamycin nanocomposites were easily dispersed into a physiological buffer, displaying a transparent solution with a slightly reddish colour.

To explore the release profile of adriamycin from the ChrGO/adriamycin complexes, the different pH values of PBS were introduced to imitate tumour cell environments. Additional file 1: Fig. S2 shows the cumulative release profiles of adriamycin from the ChrGO/adriamycin at pH 5 and 7.4. The adriamycin released from ChrGO/adriamycin was characterized by an initial fast release and then a stage of slower release in pH 5.0 PBS solution. The cumulative release of adriamycin was 6.5% in the first 3 h and then displayed a slow increase to 26.7% in 96 h at pH 5.0. By contrast, the adriamycin percentage of the ChrGO/adriamycin increased slower and was lower at pH 7.4, displaying a release percentage of adriamycin from 2.5% in the earlier 3 h to 7.4% in 96 h. Previous studies have shown the release profiles of drugs from functionalized GO very slowly in a medium at pH 7.4 [48]. The protonation of carboxyl groups on ChrGO decreases π–π stacking, H-bonding and electrostatic interactions between adriamycin and ChrGO, facilitating the release of adriamycin in the acetic medium [49]. The pH sensitivity gives ChrGO/adriamycin potential for site-specific drug delivery, which subsequently increases the cytotoxicity in tumour cells.

### Therapeutic Efficacy of ChrGO/Adriamycin Complexes

**Fig. 7:**
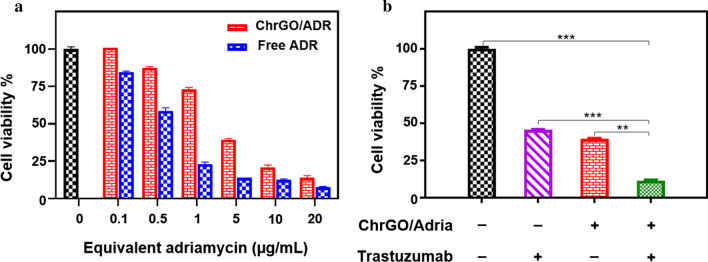
**a** In vitro cell viability of BT-474 cells incubated with concentrations of free adriamycin and adriamycin loaded in ChrGO/adriamycin complexes, **b** cytotoxicity of ChrGO/adriamycin complexes (5 μg/mL) in combination with trastuzumab (5 μg/mL) in BT-474 cells. Adria: adriamycin. **p < 0.01, ***p < 0.001

The therapeutic efficacy of the ChrGO/adriamycin complexes within HER2-overexpressing BT-474 cancer cells was investigated by the proliferation inhibition assay. Overexpression of the HER2 receptor has a key role in the transformation of BT-474 breast cancer cells. Treatment with trastuzumab alone, an anti-HER2 monoclonal antibody, results in significant growth inhibition of BT-474 cells [50]. As shown in Fig. 7a, after treatment with ChrGO/adriamycin complexes, the cell viability of BT-474 cells was decreased significantly, demonstrating a dosage-dependent toxic effect. Besides, when compared to free adriamycin, the ChrGO/adriamycin complexes demonstrated a less effective performance in killing BT-474 cells, which was ascribed to the gradual diffusion of loaded adriamycin rather than direct treatment with free adriamycin [51]. However, the therapeutic efficacy of the ChrGO/adriamycin complexes was close to that of free adriamycin as their concentration of ChrGO/adriamycin complexes increased, which can be explained by the sustained adriamycin release from the ChrGO carrier.

To study whether trastuzumab, an anti-HER2 monoclonal antibody, could improve the antitumour activity of equivalent amounts of adriamycin loaded on ChrGO, BT-474 cells were treated with trastuzumab alone or in combination with ChrGO/adriamycin. Treatment with trastuzumab alone produced a significant inhibition of BT-474 proliferation, which is consistent with a previous report [52]. The combined therapy with trastuzumab and ChrGO/adriamycin resulted in an enhanced cell growth inhibition effect, as shown by a reduction of 88.5% compared with a reduction of 54.5% with trastuzumab alone (5 μg/mL) and 59.5% with equivalent ChrGO/adriamycin alone (5 μg/mL) versus the negative control (Fig. 7b). The results suggest that the ChrGO system may be effectively used to develop composites for combination therapies, and the combined treatment of ChrGO/adriamycin and trastuzumab resulted in a modest, but significant, reduction of cell viability compared to each drug alone.

### Cell Cycle Analysis and Apoptosis

**Fig. 8:**
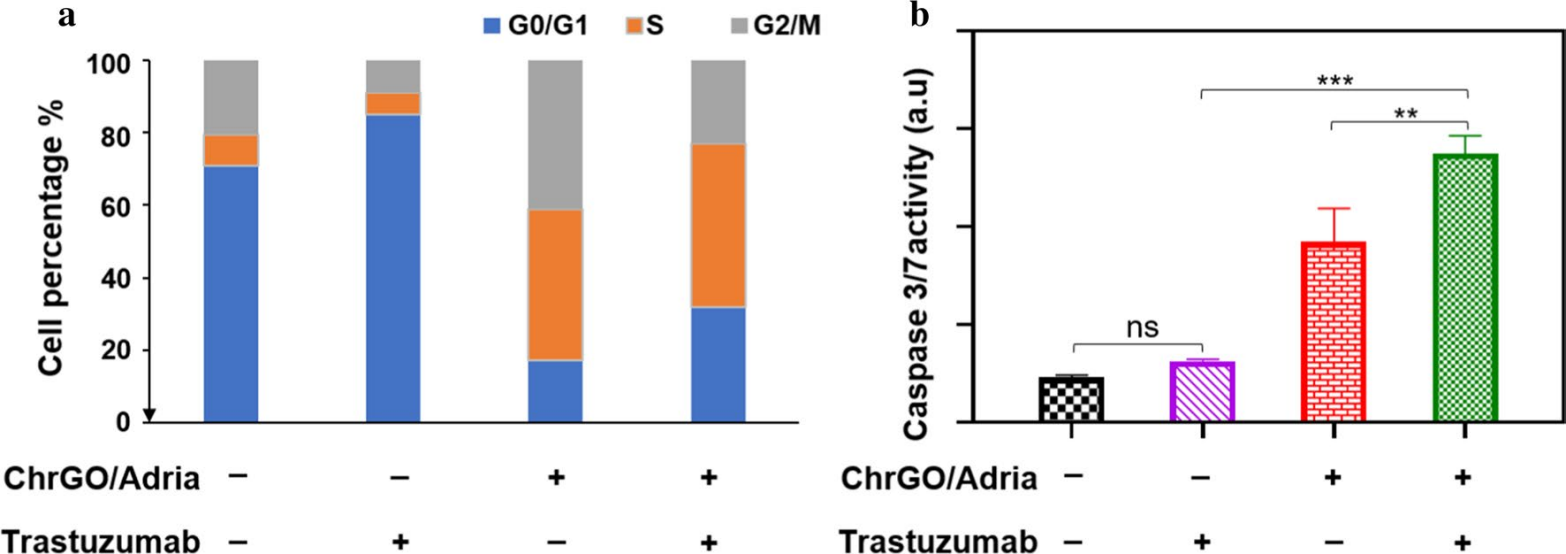
**a** Cell cycle analysis after treatment with ChrGO/adriamycin alone or in combination with trastuzumab in BT-474 cells, **b** effect of ChrGO/adriamycin (5 μg/mL) plus trastuzumab (5 μg/mL) on induction of apoptosis in BT-474 cells. Adria: adriamycin. **p < 0.01, ***p < 0.001

To determine whether the ChrGO/adriamycin complexes have effects on cell cycle progression, a cell cycle assay of ChrGO/adriamycin complexes alone or in combination treatment with trastuzumab was performed on BT474 cells. As shown in Fig. 8a, flow cytometry analysis revealed that trastuzumab alone increased the cell population in the G0/G1 phase. Treatment with ChrGO/adriamycin alone mediated a significant reduction in the number of G0/G1 cells and accumulation in S phase and G2/M phase compared to control cells. Besides, ChrGO/adriamycin combined with trastuzumab mediated S phase arrest, accompanied by a significant decrease in the number of cells in the G0/G1 phase. While ChrGO/adriamycin is most active in the S phase of the cell cycle, ChrGO/adriamycin treatment with trastuzumab can cause an increase in G0/G1 phase compared to ChrGO/adriamycin alone. A previous report showed that trastuzumab can cause cell arrest in the G1 phase [53]. Adriamycin inhibits cell proliferation and DNA replication, ultimately leading to cell cycle arrest [54].

To assess the effects of ChrGO/adriamycin on apoptotic molecules, BT-474 cells were treated for 48 h with either ChrGO/adriamycin, trastuzumab, or a combinational treatment of both agents. For the visualization of apoptosis, caspase 3/7 activity has been extensively used as an apoptosis-specific marker due to its activity related to the process of apoptosis [55]. As observed from Fig. 8b, compared to the negative control, treatment with ChrGO/adriamycin (5 μg/mL) alone significantly increased the caspase 3/7 activity. However, trastuzumab (5 μg/mL) alone did not significantly increase caspase 3/7 expression, suggesting that trastuzumab does not induce apoptosis. In contrast, treatment with ChrGO/adriamycin (5 μg/mL) plus trastuzumab (5 μg/mL) significantly increased caspase 3/7 activity compared to that with each drug alone. The dual-targeted therapy showed higher apoptosis, indicating superior therapeutic efficacy due to the presence of different mechanisms of action. Similar to other studies, adriamycin amplifies the apoptotic response in HER2-overexpressing cancer cells [56]. In conclusion, ChrGO/adriamycin combined with trastuzumab induces cell cycle arrest and apoptosis, which ultimately results in augmented cell death.

## Conclusions

In the present work, the chitosan-functionalized graphene oxide nanosheets were structured with microwave-assisted reduction, which demonstrated biocompatibility and good dispersion stability. The as-prepared nanocomposites showed high efficiency of drug encapsulation and delivery. The ChrGO/adriamycin nanosheets displayed significant growth inhibition of BT-474 in a dose-dependent manner. The combined treatment of ChrGO/adriamycin and trastuzumab resulted in superior therapeutic efficacy in BT-474 cells compared to that with each agent alone. The results are favourable for the development of intracellular nanocarriers to deliver drugs in a controlled manner, which is expected to improve the therapeutic effect on HER2-overexpressing cancer therapies.

## Supplementary Information

**Additional file 1.** **Supplemental Fig. S1.** Zeta potential of GO and ChrGO. **Fig. S2.** The release of loaded adriamycin at different pH values.

## Data Availability

The datasets supporting the conclusions of this current study are available from the corresponding authors upon reasonable request.

## Abbreviations

ChrGO: Chitosan-functionalized graphene oxide
EGFR: Epidermal growth factor receptor
HER2: Human epidermal growth factor receptor-2
GO: Graphene oxide
DMEM: Dulbecco's modified Eagle medium
FBS: Fetal bovine serum
TEM: Transmission electron microscopy
UV–Vis: Ultraviolet–visible
FTIR: Fourier transform infrared
XRD: X-ray diffraction
XPS: X-ray photoelectron spectroscopy
TGA: Thermogravimetric analysis
PBS: Phosphate-buffered saline
SAED: Selected-area electron diffraction
TGA: Gravimetric analysis
