# Supplementary material for: Microwave-Assisted Chitosan-Functionalized Graphene Oxide as Controlled Intracellular Drug Delivery Nanosystem for Synergistic Antitumour Activity
Source: Nanoscale Res Lett. 2021 Apr 30;16:75. doi: 10.1186/s11671-021-03525-y (PMC8087749; doi:10.1186/s11671-021-03525-y)
Supplement: Supplementary file 1 — Additional file 1. Supplemental Fig. S1. Zeta potential of GO and ChrGO. Fig. S2. The release of loaded adriamycin at different pH values. [file 11671_2021_3525_MOESM1_ESM.docx]

**Microwave-Assisted Chitosan-Functionalized Graphene Oxide as Controlled Intracellular Drug Delivery Nanosystem for Synergistic Antitumor Activity**

Mengjun Shu^1^, Feng Gao^1^, Min Zeng^1^, Chulang Yu^5^, Xue Wang^4^, Renhua Huang^6^, Jianhua Yang^1^, Yanjie Su^1^, Nantao Hu^1^, Zhihua Zhou^1^, Ke Liu^4^*, Zhi Yang^1^*, Hongtao Tan^2^*, Lin Xu^3^*

^1^ Key Laboratory of Thin Film and Microfabrication (Ministry of Education), Department of Micro/Nano Electronics, School of Electronic Information and Electrical Engineering, Shanghai Jiao Tong University, Shanghai 200240, People’s Republic of China.

^2^ Department of General Surgery, The First Affiliated Hospital of Harbin Medical University, Harbin 150001, People’s Republic of China.

^3^ Department of Ophthalmogy, Shanghai General Hospital (Shanghai First People's Hospital), School of Global Health, Chinese Center for Tropical Diseases Research, Shanghai Jiao Tong University School of Medicine; Shanghai Eye Disease Prevention & Treatment Center/Shanghai Eye Hospital; National Clinical Research Center for Eye Diseases; Shanghai Key Laboratory of Ocular Fundus Diseases; Shanghai Engineering Center for Visual Science and Photomedicine, Shanghai 200080, People’s Republic of China.

^4^ Department of Dermatology, Shanghai Ninth People’s Hospital, Affiliated to Shanghai Jiao Tong University School of Medicine, Center for Specialty Strategy Research of Shanghai Jiao Tong University China Hospital Development Institute, Shanghai 200011, People’s Republic of China.

^5^ State Key Laboratory for Managing Biotic and Chemical Threats to the Quality and Safety of Agro-products, Key Laboratory of Biotechnology in Plant Protection of MOA and Zhejiang Province, Institute of Plant Virology, Ningbo University, Ningbo 315211, People’s Republic of China.

^6^ Department of Radiation, Renji Hospital, School of Medicine, Shanghai Jiao Tong University, Shanghai 200240, People’s Republic of China.

Correspondence: tanhongtao2013@163.com, qq593783531@163.com, zhiyang@sjtu.edu.cn, linxu@sjtu.edu.cn


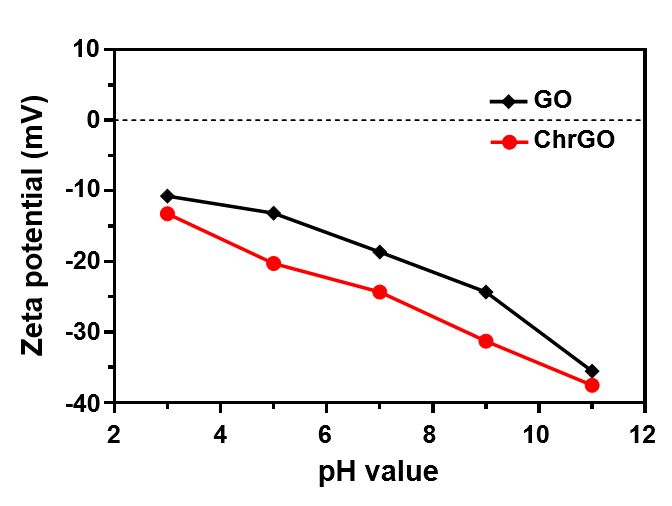


**Fig. S1** Zeta potential of GO and ChrGO


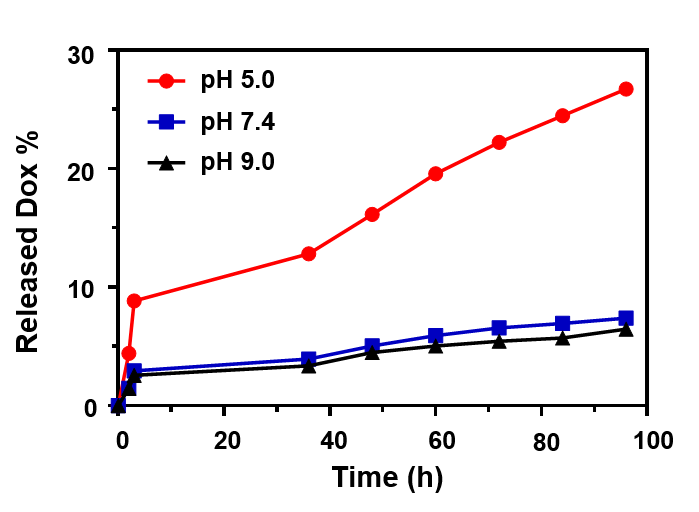


**Fig. S2** The release of loaded adriamycin at different pH values.
